# Two case reports of desensitization in patients with hypersensitivity to iron

**DOI:** 10.1186/s40413-017-0166-z

**Published:** 2017-10-10

**Authors:** Edgardo Chapman, Drixie Leal, Leidy Alvarez, Mónica Duarte, Elizabeth García

**Affiliations:** 1 Fundación Santa Fe de Bogota, Carrera 7 N 117–15, Bogotá, Colombia; 2 University of Cartagena, Cra. 6 #36, Cartagena, Bolívar, Colombia; 3 Faculty of Medicine University of the Andes, Carrera 1 No 18 A –, 10 Bogotá, Colombia

**Keywords:** Iron deficiency anemia, Hypersensitivity, Intravenous iron allergy

## Abstract

**Background:**

Iron deficiency anemia is a disease that can significantly compromise a patient’s quality of life. Desensitization is a safe and effective treatment option for iron-deficient anemic patients who require intravenous iron despite their hypersensitivity to iron. This report describes a safe desensitization protocol for patients with iron hypersensitivity who require iron treatment for their clinical improvement.

**Case presentation:**

Two patients of 20 and 46-year-old diagnosed with secondary iron deficiency anemia hipermenorreas and a clinical history of fail treatment with oral iron, who presented a reaction of the anaphylactic type while they receive iron parenteral sucrose. Therefore, the patients were treated with the desensitization protocol applied for patients with hypersensitivity to iron.

**Conclusion:**

Iron deficiency anemia is a disease that can significantly compromise the quality of life of patients. The desensitization protocol for patients with hypersensitivity to iron is a safe and effective treatment option for patients with a history of allergy to intravenous iron. This case report shows the usefulness to use the desensitization protocol for patients with hypersensitivity to iron.

## Background

Iron deficiency anemia is a disease that attracts worldwide interest, not only because of its links to common instances of malnutrition but also because of the severely compromised quality of life of those who suffer from its symptoms. An OMS study conducted between 1992 and 2005 estimated that the worldwide prevalence of anemia was 24.8%, of which 50% were cases of iron deficiency anemia. A recent meta-analysis published by Kassebaum and colleagues reviewed the prevalence of anemia worldwide from 1990 and 2010 and found that more than 30% of the global population suffered from anemia and that iron deficiency was the primary cause [1].

Currently, iron treatment is a standard procedure and is considered a safe and effective remedy. However, instances of severe allergic reactions have occurred following iron administration, irrespective of the means of delivery.

Twenty-five percent of all adverse reactions are known to result from iron hypersensitivity, which, although rare, can prove fatal. This same scarcity has made it difficult to accurately determine the rate of occurrence and attendant risks, although it is estimated that 1 in every 5 million doses of intravenous iron applied produce allergic reactions, with a mortality rate in the US of 3 deaths per year [2, 3].

Parenteral iron therapy is the current solution for patients who fail to complete a course of oral iron therapy due to either intolerance or suspension of the treatment. Similarly, parenteral iron therapy is used if symptoms persist or worsen, in cases of intestinal malabsorption (for example, inflammatory bowel disease or previous gastrointestinal surgery), in the presence of peptic ulcers and active bleeding, as well as in cases of any postoperative impediment to oral ingestion. Other reasons to use parenteral iron therapy could include perioperative anemia, functional iron deficiency, and treatment with erythropoiesis-stimulating agents. Parenteral iron is also used for nephrology patients, those suffering from malignancies or undergoing chemotherapy, and patients who are either pregnant or postpartum [4].

Currently, a standardized desensitization protocol for intravenous iron sucrose is not available at our institute. This report describes two cases of patients who suffered reactions to the intravenous administration of iron and who then underwent desensitization to intravenous iron according to the protocol illustrated in another case report previously described [3, 5, 6].

## Case presentation 

### Case 1

A 20-year-old patient who complained of hypermenorrhea was diagnosed with secondary iron deficiency anemia**.** She was treated by the hematology and gynecology departments for one year and underwent hormonal therapy and a course of oral iron supplements. The hypermenorrhea was controlled, but iron deficiency persisted, along with a gastrointestinal intolerance to the drug (gastritis and constipation). Treatment with parenteral iron sucrose was ordered; however, after only 10 min of the new treatment, the patient experienced an anaphylactic reaction that included abdominal pain, skin lesions or hives, angioedema and dysphonia.

### Case 2

A 46-year-old patient was diagnosed with iron deficiency anemia caused by hypermenorrhea and uterine fibroids and accompanied by gastrointestinal absorption disorders and vitamin B12 deficiency. She received a course of oral iron treatment for approximately 2 years without presenting a hematologic response (Table 1). However, when the intravenous iron sucrose treatment was initiated, the patient presented hives, angioedema, back pain and dyspnea after 5 min.

**Table 1:** Laboratory test results on admission

Case	HB g/dL	HTO %	MCV fL	MCH pg	MCHC g/dL	Ferritin u/L	Iron u/dL
1	12.9	39	90.7	30.1	33.2	6.4	70
2	9.8	32	74.5	22.7	30.5	5.5	50

*HB* hemoglobin, *HTO* hematocrit, *MCV* mean corpuscular volume, *MCH* mean corpuscular hemoglobin, *MCHC* mean corpuscular hemoglobin concentration

In both cases, the persistence of anemia negatively affected each patient’s quality of life, resulting in intense fatigue, weakness, and dizziness. Parenteral iron was prescribed, and iron sucrose desensitization treatment was performed.

### Protocol

The desensitization protocol began with 48 h of hospitalization to prepare for the procedure through the provision of systemic steroids, antihistamines and anti-leukotrienes (Table 2). The patients were admitted to the intensive care unit under continuous monitoring, and an hour before starting the procedure, 80 mg of methylprednisolone and 25 mg of hydroxyzine were administered intravenously. A total of 10 iron sucrose doses were administered with increasing concentrations, at 15-min intervals. The procedure started with an intravenous iron sucrose supply of 0.1 mg, and the complete cumulative dose was 100 mg (Table 3).

**Table 2:** Treatment prior to the procedure

Drug name	Presentation	Dose administered Via/hous
Prednisone	50 mg tablets	50 mg orally every 24 h
Cetirizine	10 mg tablets	10 mg orally every 12 h
Montelukast	10 mg tablets	10 mg orally every 12 h

**Table 3:** Desensitization protocol for parenteral iron

Number of dosages	mg delivered	Amount ml	Time minutes
1	0.1	0.1	15
2	0.2	0.2	15
3	0.5	0.5	15
4	1	1	15
5	2	2	15
6	5	5	15
7	10	10	15
8	20	20	15
9	50	50	15
10	100	100	30

 In both cases, the procedure was completed without complications. Oral courses of cetirizine (10 mg every 12 h) and montelukast (10 mg every 12 h) were continued daily for the duration of the hospitalization, and daily administration of iron continued in equal doses until completion of the estimated deficit for each patient.

A total supply of 1400 mg of intravenous iron sucrose was required to complete the treatment in each of the two cases. The daily supply of 200 mg was administered intravenously in the following manner: 20 mg was injected in two doses over the first 30 min while the patient was monitored for a reaction. In the absence of further reaction, the remaining 180 mg of the daily dose was administered over a period of 4 h, with time intervals between each daily dose of 24 to 26 hours [7].

No secondary allergic reactions were noted in either case.

The patients’ follow-up examinations demonstrated evidence of the success of the treatment, and the patients reported the total disappearance of their symptoms (Table 4).

**Table 4:** Clinical follow-up examinations at two months

Case	HB g/dL	HTO %	MCV fL	MCH pg	MCHC g/dL	Ferritin u/L	Iron u/dL
1	13.9	41	88.8	30.2	34	286.5	99
2	13.9	41.8	89.2	29.2	32.3	98.7	75

*HB* hemoglobin, *HTO* hematocrit, *MCV* mean corpuscular volume, *MCH* mean corpuscular hemoglobin, *MCHC* mean corpuscular hemoglobin concentration

## Discussion

The International Consensus on Drug Allergy (ICON) defines drug hypersensitivity reactions (DHRs) as those arising from the activation of the adaptive immune system after the administration of a drug. Clinically, these reactions are divided into immediate DHRs (occurring within the first hour after administration, possibly mediated by IgE) or non-immediate (occurring after the first hour, T-cell dependent) [8].

Various factors have been identified that increase both the risk of hypersensitivity to the iron treatment and the severity of the reaction. These factors include the following: a history of previous reactions to intravenous iron, a rapid rate of administration, severe heart or respiratory disease, mastocytosis, older age, beta-blockers or ACE inhibitors, severe asthma or eczema [7].

A greater risk exists of allergic reactions to intravenous iron than to orally administered iron. This increased risk occurs because the medicine introduced directly into the blood stream does not pass through the hepcidin-ferroportin system in the intestines, which would otherwise protect against overdoses and regulate the metabolic processing of elemental iron to ensure that it is released slowly during erythropoiesis [2, 9].

Hypersensitivity reactions can be recognized based on the signs and symptoms of the patient. These could range from mild, such as itching, flushing, hot flashes, hypertension, back or joint pain, mild coughing, chest tightness, mild dyspnea, tachycardia and hypotension, to severe, including anaphylaxis. The initial reaction could worsen quickly, generating a high risk of mortality [7].

The desensitization protocol for iron sucrose has been applied in various clinics, and new opinions regarding its implementation have emerged over the years based on scientific evidence and case reviews. Initially, the use of corticosteroids and antihistamines during premedication was recommended at the commencement of treatment; these adjuvants reduce the rate of mild to moderate reactions in patients with a history of hypersensitivity. In many case reports, such as the present cases, the outcome was successful. [6, 10, 11]

However, several authors have suggested that these antihistamines should not be administered because they could cause adverse effects that could be misinterpreted as DHRs, such as a slight urticariform reaction [12–15]. Moreover, some authors suggest that corticosteroids have a greater influence on anaphylaxis than on DHRs; therefore, their usefulness in the initial part of the protocol should be reassessed [12]. Other authors have suggested that premedication should be omitted completely, although such modifications to the protocol are still being investigated [7].

Regarding the administration of the drug post-desensitization, the doses should continue to be divided into two fractions (10% followed by 90%), even after the first day of the treatment, with the aim of monitoring and ensuring the patient’s safety at each stage. This precautionary measure has been undertaken by other doctors previously [6, 11]. However, the latest recommendations of the EMA Committee for Medicinal Products for Human Use (CHMP) concerning the management of the risk of allergic reactions to intravenous iron suggest that patients should be closely monitored during the first 30 min after the application of the drug. However, it is no longer advised that fractional doses of iron be administered in the days following desensitization, as the initial clinical response of the patient to the introduction of a percentage of the dose of the drug is not considered a predictor of possible reactions to the total dose, as administered in this and other protocols [16].

Two cases of patients with anaphylactic reactions to parenteral iron sucrose have been presented in which an anaphylactic reaction occurred after the first contact with the drug but the drug was essential for treatment of the disease. Implementation of a desensitization protocol was required due to the lack of response to oral treatment. The desired result of desensitization was achieved in considerably less time than in other protocols [10, 11].

The patients were informed that drug desensitization induces a transient, immunological tolerance lasting a maximum of 48 h after the final dose is given. It is important to clarify that if a need exists to use the drug at a later date, then the procedure must be repeated.

## Conclusion

This report reflects the utility of using a desensitization protocol in patients with hypersensitivity to iron when it has not been possible to recover a normal hemoglobin level, restore mean corpuscular volume and replenish iron deposits by other treatments.

The specific use of this 10-step protocol, compared with other protocols, reduces the time of strict medical supervision that each step requires, as well as the costs derived from producing a greater number of dilutions of the drug.

However, the questions regarding premedication presented by other authors show the need to develop and apply innovative protocols in which the drugs in question are adjusted or the desensitization is performed without any premedication to increase patient safety and to build a new consensus on treatment.

## Abbreviations

HB: Hemoglobin
HTO: Hematocrit
MCH: Mean corpuscular hemoglobin
MCHC: Mean corpuscular hemoglobin concentration
MCV: Mean corpuscular volume
WHO: Word Health Organization
